# Botulinum Neurotoxin-A Inhibits Tumor Growth in a Triple-Negative Breast Cancer Preclinical Model

**DOI:** 10.3390/toxins18050212

**Published:** 2026-04-30

**Authors:** Evoli N. Lopez, Guadalupe Delgado-López, Paola Maycotte, Pablo Hernández-Jáuregui, Irma Herrera-Camacho, Nora Hilda Rosas-Murrieta, Eunice López-Muñoz, Claudia Teresita Gutiérrez-Quiroz, Uriel Ramírez-Carrera, Cindy Bandala, Lourdes Millán-Pérez-Peña, Maricruz Anaya-Ruiz

**Affiliations:** 1 Laboratorio de Bioquímica y Biología Molecular, Centro de Química del Instituto de Ciencias (ICUAP), Benemérita Universidad Autónoma de Puebla, Puebla 72570, Mexico; qfb.evolilopez@gmail.com (E.N.L.); irma.herrera@correo.buap.mx (I.H.-C.); nora.rosas@correo.buap.mx (N.H.R.-M.); lourdes.millan@correo.buap.mx (L.M.-P.-P.); 2 Laboratorio de Biología Celular del Cáncer, Centro de Investigación Biomédica de Oriente, Instituto Mexicano del Seguro Social, Puebla 74360, Mexico; delgadoliy@yahoo.com.mx (G.D.-L.); pablohja44@gmail.com (P.H.-J.); 3 Laboratorio de Bioquímica Metabólica, Centro de Investigación Biomédica de Oriente, Instituto Mexicano del Seguro Social, Puebla 74360, Mexico; bisbenzimida@gmail.com; 4 Unidad de Investigación Médica en Medicina Reproductiva UMAE Hospital de Gineco Obstetricia No. 4, Luis Castelazo Ayala, Instituto Mexicano del Seguro Social, Mexico City 01090, Mexico; astridkaryme2001@yahoo.com.mx; 5 Departamento de Anatomía Patológica, Centro Médico Nacional “General de División Manuel Ávila Camacho”, Hospital de Especialidades, Instituto Mexicano del Seguro Social, Puebla 72089, Mexico; claudia.gutierrez@imss.gob.mx (C.T.G.-Q.); hturc212@gmail.com (U.R.-C.); 6 Laboratorio de Neurociencia Traslacional en Enfermedades Crónicas y Emergentes, Escuela Superior de Medicina, Instituto Politécnico Nacional, Mexico City 11340, Mexico; crodriguezba@ipn.mx

**Keywords:** TNBC, botulinum neurotoxin A, SV2A, drug repurposing, preclinical model, immune system, 4T1

## Abstract

Triple-negative breast cancer (TNBC) continues to be a medical challenge requiring multiple treatment options. SV2A, a protein involved in vesicular release, has emerged as a promising biomarker for various cancers. Research shows that botulinum neurotoxin type A (BoNT/A), which binds to SV2A, the BoNT/A receptor, can inhibit the growth of prostate and breast cancer cells, suggesting its potential as an alternative treatment for breast cancer. The purpose of this study was to determine the potential of BoNT/A to inhibit tumor growth in a mouse preclinical model. BoNT/A was evaluated for its effects in an in vitro model employing 4T1 cells and in an in vivo model of orthotopically inoculated 4T1 cells in BALB/c mice. BoNT/A inhibited the proliferation of 4T1 cells, which express the SV2A protein; decreased tumor growth in the preclinical model; and decreased inflammation, associated with fewer blood neutrophils and monocytes, suggesting an immunomodulatory and anti-inflammatory effect. The effect of BoNT/A on the TNBC model supports its use as a repurposed drug for this type of aggressive cancer. Our results emphasize the significance of the SV2A receptor and its interaction with BoNT/A as promising therapeutic targets, particularly for TNBC.

## 1. Introduction

Triple-negative breast cancer (TNBC) is a highly aggressive subtype of breast cancer with limited treatment options and poor clinical outcomes. It represents 15–25% of breast cancer cases, and it is identified by the absence of progesterone, estrogen, and HER2 receptors [1,2]. Available treatment options for TNBC are largely limited to surgery, adjuvant chemotherapy [3], and radiotherapy [4]. Nevertheless, many patients are diagnosed at an advanced stage, when the opportunity for surgical intervention has already passed. Despite extensive research efforts, the number of drugs approved for TNBC remains limited [5]. The current approved targeted drugs for this cancer type include pembrolizumab, a humanized monoclonal antibody that blocks PD-1 that has been indicated in combination with chemotherapy for the treatment of high-risk early-stage TNBC, and for the treatment of locally recurrent unresectable or metastatic TNBC expressing PD-L1 [6,7]. For this latter group, atezolizumab, a PD-L1 inhibitor, has also been tested [8,9]. Sacituzumab govitecan, a monoclonal antibody against TROP-2, is a drug approved by the U.S. Food and Drug Administration (FDA) for the treatment of metastatic TNBC in patients who have already undergone at least two prior treatments for metastatic disease [10]. Also, PARP inhibitors (Oloparib and Talazoparib) have been approved for patients with germline BRCA mutations and metastatic disease [11]. And finally, trastuzumab deruxtecan has been approved for HER2-low breast cancer, but since some TNBC patients may exhibit a HER2-low phenotype, they might be eligible for this drug based on biopsy results [12]. However, despite the efficacy of these drugs, their use is sometimes limited to metastatic disease or to limited patients, and they have been reported to have adverse effects, such as anemia and neutropenia, which increase with chemotherapy. Therefore, the development of new drugs for the potential treatment of TNBC is necessary.

Botulinum neurotoxin-A (BoNT/A) is a zinc endopeptidase with a 150 kDa molecular weight produced by the bacterium *Clostridium botulinum*, which has antitumor effects in various types of cancer [13]. BoNT/A decreases the proliferation, through apoptosis, of various cancer cells, including prostate (LNCaP) and breast cancer (T47D) cell lines, through a mechanism dependent on the interaction with the synaptic vesicle glycoprotein 2 (SV2) [9,10,11,12]. Furthermore, other researchers have seen the antitumor effect of the toxin in preclinical models of prostate [14], pancreatic [15], and colon cancer [16], or in a hepatocellular carcinoma model [17].

Currently, BoNT/A is included in several clinical trials for the treatment of pain in cancer patients [18,19,20,21], with its analgesic properties initially documented in 1986 in patients with cervical dystonia [22]. Currently, chronic migraine is the only condition where its indication has been approved for clinical use; however, multiple systematic reviews of clinical trials have provided evidence supporting BoNT/A efficacy across diverse pain conditions [23] and for the treatment of stomach cancer [24]. Regarding breast cancer, it has been described that a single administration of BoNT/A reduces the pain of patients after breast cancer surgery, preserving the effect for up to 6 months [2]. However, there have been no studies involving BoNT/A in the treatment of TNBC. Therefore, the present study aims to determine the potential use of BoNT/A for the treatment of TNBC.

Experimental animal models are essential tools for unraveling the physiological mechanisms, disease progression, and potential therapeutic approaches in breast cancer research [25]. Among these, the syngeneic TNBC model established by inoculating BALB/c mice with 4T1 cells closely replicates the clinical characteristics of stage IV breast cancer in humans [26]. Previous comparative analyses demonstrate that SV2A from human, mouse, and rat share a high level of amino acid sequence homology, particularly in the luminal and transmembrane regions relevant to BoNT/A binding. This supports the validity of comparing SV2A expression between human and mouse cells in our study [27]. Notably, this model enables the study of immune system dynamics, offering a distinct advantage over traditional xenograft models in immunosuppressed hosts, which are commonly employed for human cancer cell xenograft studies [28].

The involvement of vesicular release proteins in breast cancer progression remains largely unexplored. Nonetheless, elevated expression levels of the three isoforms of synaptic vesicle protein 2 (SV2A, SV2B, and SV2C) have been observed in multiple human breast cancer cell lines—namely MDA-MB-231 (triple-negative), SKBR3 (HER2-positive), and T47D (PR-positive and ER-positive)—in comparison with MCF-10A non-tumorigenic cell line [29]. Studies of breast cancer-patient biopsies have revealed overexpression of SV2 A, B, and C isoforms in tumor tissues in comparison with normal breast tissue, and their expression was related to different clinicopathological features. In addition, differences were observed in the expression of these isoforms with regard to metastasis [30]. BoNT/A has also been shown to markedly downregulate SV2A expression in various breast cancer cell lines, including T47D, MDA-MB-231, and MDA-MB-453 [31]. Notably, BoNT/A exhibited greater cytotoxic activity in T47D cells compared with normal MCF10A cells, and BoNT/A induced a caspase-3- and -7-dependent apoptotic process in these tumor cells [32]. Complementary findings suggest that BoNT/A potentiates the therapeutic efficacy of Herceptin in SK-BR-3 and BT-474 breast cancer cell lines [33]. Therefore, targeting the SV2 receptor with BoNT/A appears as a promising therapy for cancer, warranting its study on challenging cancer types like TNBC.

## 2. Results

### 2.1. SV2A Is Overexpressed in a Mouse TNBC Cell Line and Is Sensitive to BoNT/A Treatment

#### 2.1.1. SV2A Is Present and Overexpressed in the Mouse 4T1 TNBC Cell Line

A comparative assay was performed in four cell lines to evaluate SV2A receptor expression: human glioblastoma U87 cells; a human TNBC cell line, MDA-MB-231, as a positive control; the murine TNBC cell line 4T1 to determine the presence and relative abundance of the receptor; and the non-tumoral murine mammary epithelial cell line EpH4-ev, used as a reference control.

**Figure 1:**
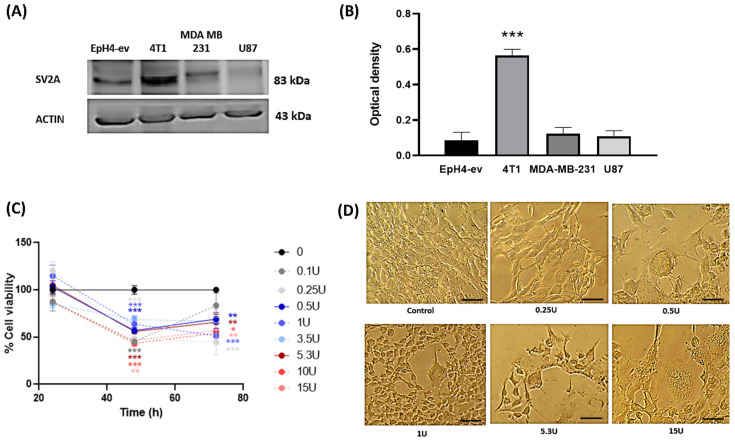
SV2A levels are high in the 4T1 murine breast cancer cell line, and this cell line is sensitive to BoNT/A treatment. (**A**) SV2A protein levels (83 kDa) in EpH4-ev (normal mouse mammary cells), 4T1 (mouse TNBC cells), MDA MB 231 (human TNBC cells), and U87 (human glioblastoma cells); β-actin was included as a loading control. (**B**) Normalized SV2A optical density from the Western blot. Graph shows mean +/− SEM, one-way ANOVA, post hoc Tukey *** *p* < 0.05, of three independent experiments. (**C**) Evaluation of cell viability at 48 and 72 h on the 4T1 cell population. Two-way ANOVA, post hoc Tukey * *p* < 0.5, ** *p* < 0.1, and *** *p* < 0.01. (**D**) Representative images show the effect of different doses of BoNT/A on cell morphology of 4T1 breast cancer cells at 72 h (optical microscopy, Nikon TMS, Nikon, NY, USA) 20×. Scale bar: 100 μm.

Western blot analysis revealed SV2A expression in all the cell lines tested, with an expected molecular weight of approximately 83 kDa. Notably, a marked overexpression of SV2A was observed in the 4T1 breast cancer cells from BALB/c mice (Figure 1A). Densitometric analysis demonstrated that SV2A expression in 4T1 cells was up to three-fold higher compared with the non-tumoral EpH4-ev cells and the other cancer cell lines in which SV2A expression had been previously described. This difference was statistically significant (Figure 1B).

These findings demonstrate the presence and overexpression of SV2A in murine TNBC 4T1 cells when compared to a mouse non-tumorigenic control, probably suggesting a role for this receptor in the development, progression, and/or metastasis of TNBC.

#### 2.1.2. The 4T1 Cell Line Was Sensitive to BoNT/A Treatment

A BoNT/A dose–response analysis was performed in the 4T1 murine breast cancer cell line. BoNT/A induced a slight, non-significant decrease in viability (20%) only at the maximum dose used (15 U) at 24 h (data not shown). At 48 h and 72 h, viability progressively decreased across multiple concentrations (0.25–15 U), indicating a clear effect on cell viability (Figure 1C). The calculated half-maximal effective concentrations (EC50) were 16.28 U at 24 h, 1.25 × 10^−7^ U at 48 h, and 1.71 × 10^−7^ U at 72 h (Figure 1C). Morphological alterations were also observed at 72 h in BoNT/A-treated 4T1 cells, including reduced cell size, detachment, vacuolization, and the appearance of filamentous phenotypes (Figure 1D).

These results demonstrate that BoNT/A exerts a reduction in viability in 4T1 cells, supporting its relevance as a preclinical tool in the study of TNBC.

### 2.2. In Vivo Results

#### 2.2.1. BoNT/A Decreased Tumor Growth in a Preclinical Model of TNBC

**Figure 2:**
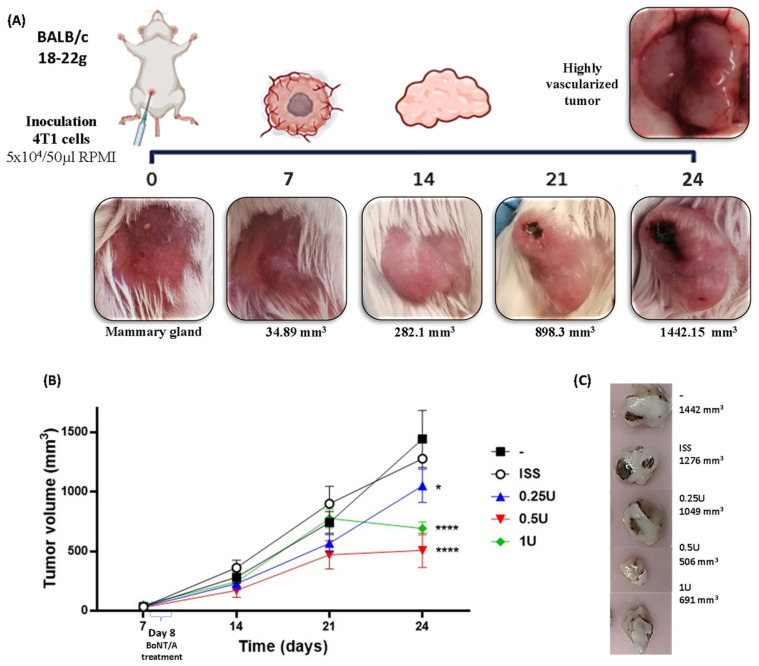
BoNT/A treatment decreased tumor growth in a syngeneic, orthotopic TNBC tumor model. (**A**) Cells were inoculated into the fourth nipple of the mouse (day 0); representative pictures of tumors from the different days are shown with the average tumor volume in control mice (day 7, 34 ± 9 mm^3^; day 14, 282 ± 83 mm^3^; and day 21, 898 ± 125 mm^3^). Epithelial necrosis was observed starting on day 21, increasing until day 24 (1442.15 ± 241 mm^3^), when a highly irrigated tumor was observed post-sacrifice. (**B**) Tumor development with BoNT/A treatment (ISS, isotonic saline solution). Tumors were monitored from day 7 until day 24. Tumors with 0.25 U, 0.5 U, and 1 U BoNT/A had statistical differences to control mice decreasing by approximately half their size. (**C**) Evidence of tumor growth on the day of sacrifice, on day 24, when control, ISS, and 0.25 U BoNT/A treated-tumors presented epithelial necrosis. Graph shows mean +/− SEM of 6 mice per group. Two-way ANOVA, Tukey’s post hoc * *p* < 0.05; **** *p* < 0.0001.

In this study, orthotopic administration of the 4T1 cells induced tumor development within 24 days, with day 0 defined as the day of inoculation. Over time, the macroscopic characteristics of the mammary tumors generated in BALB/c mice showed a nodular morphology located on the fourth nipple, multilobulated with well-defined margins, smooth surface, pale pink color, and increased consistency. The tumors were highly vascularized, and, as they enlarged, ulceration occurred with extension toward the inguinal and upper thoracic regions. Marked vascularization was evident from day 7, when the average tumor volume reached 34 ± 10 mm^3^ (Figure 2A).

Treatment was administered as a single dose of BoNT/A at three different concentrations (0.25 U, 0.5 U, and 1 U) on day 8. Post-treatment analysis showed that animals receiving the lowest dose (0.25 U BoNT/A) reached a final tumor volume of 1049 ± 144 mm^3^, similar to the untreated tumor group (1442 ± 225 mm^3^) and the saline-treated group (1276 ± 36 mm^3^). In contrast, animals treated with 0.5 U BoNT/A exhibited a marked reduction in tumor growth (506 ± 145 mm^3^), while those receiving 1 U BoNT/A also showed a significantly smaller tumor size (691 ± 51 mm^3^). Thus, BoNT/A at doses of 0.5 U and 1 U reduced tumor volume by approximately 50%, with a difference of 770 cm^3^ and 585 cm^3^, respectively, compared with saline-treated groups (Figure 2B).

Notably, during handling, animals treated with BoNT/A showed improved tolerance to handling compared with the tumor and tumor + ISS (saline) groups, particularly after day 18, when tumor burden became substantial. Furthermore, histological analysis revealed reduced necrotic areas in tumors treated with BoNT/A at the 0.5 U and 1 U doses (Figure 2C).

#### 2.2.2. BoNT/A Treatment Did Not Affect the General Health of Mice with or Without TNBC Tumors

The animals were monitored throughout the experiment, and statistically significant differences in body weight were observed between the control groups (with and without BoNT/A) and the tumor-bearing groups (with and without BoNT/A). In other words, tumor-bearing animals exhibited weight loss, indicating a deterioration in their clinical condition, while BoNT/A treatment did not affect this parameter (Supplementary Figure S1A). Moreover, non-tumor-bearing animals did not show a weight decrease at the 0.5 U BoNT/A dose, suggesting a better tolerability of BoNT/A at this dose.

This observation was further supported by daily food intake measurements. Animals in the control groups consumed approximately 4 g/day, whereas those in the tumor-bearing groups, with or without BoNT/A treatment, consumed approximately 3.5 g/day (Supplementary Figure S1B). Importantly, healthy mice without tumor treated with BoNT/A (0.25 U, 0.5 U, or 1 U) consumed more food than tumor-bearing mice treated with saline or the lowest and highest dose of BoNT/A. However, tumor-bearing mice treated with 0.5 U BoNT/A did not decrease their food consumption when compared to healthy mice with BoNT/A treatment, suggesting a better tolerability and probably less side effects of this dose of BoNT/A.

#### 2.2.3. BoNT/A Decreased Inflammation in the Preclinical Model of TNBC

**Figure 3:**
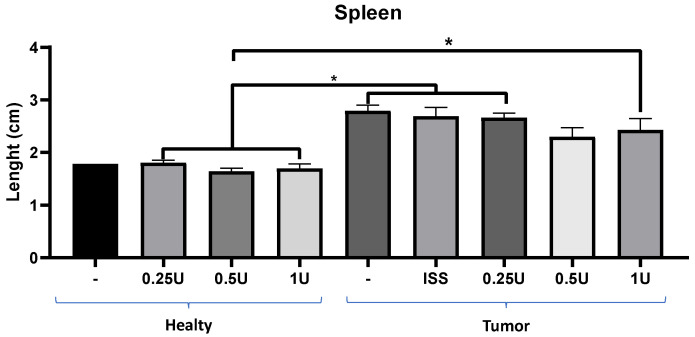
Effect of BoNT/A on splenomegaly in healthy mice compared to those with breast cancer. Effect of BoNT/A on spleen size. Tumor-bearing animals treated with 0.5 U BoNT/A did not increase their spleen size when compared to untreated tumor-bearing mice. No significant changes in spleen size were observed in healthy animals following treatment. Graphs show mean +/− SEM of 3–6 mice; one-way ANOVA, Tukey’s post hoc, * *p* < 0.05.

In this preclinical model, statistically significant differences were found between healthy animals and tumor-bearing animals, even in those treated with 0.25 U or 1 U BoNT/A (Figure 3). Interestingly, although non-significant, a reduction in spleen size was apparent in the treatment group with 0.5 U BoNT/A that also exhibited decreased tumor growth. These findings suggested that BoNT/A could be exerting an immunomodulatory effect on splenic cells in tumor-bearing animals. It is important to note that the healthy animals maintained a normal spleen size, both with and without toxin administration, as shown on Figure 3.

To further evaluate the effect of BoNT/A on blood parameters, a hematological analysis was performed. It is well established that neutrophil elevation reflects infection or inflammation, as these cells target foreign agents, and previous studies have shown increased inflammation in the 4T1 TNBC model [34].

**Figure 4:**
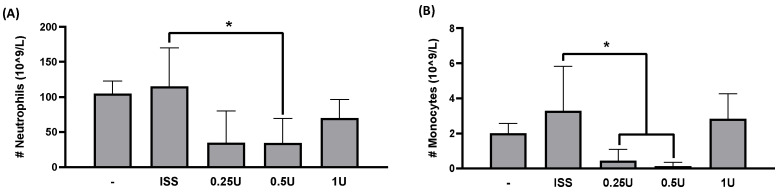
BoNT/A effect on immune response reflected in neutrophil and monocyte reduction in a peripheral blood analysis in the breast cancer preclinical model. (**A**) The number of circulating neutrophils decreased in the 0.5 U BoNT/A-treated tumor-bearing mice when compared to their respective ISS control. (**B**) The monocytes number decreased with 0.25 U and 0.5 U BoNT/A. (**A**,**B**) Graphs show mean +/− SEM of 6 independent experiments; one-way ANOVA, Tukey’s post hoc, * *p* < 0.05.

Remarkably, BoNT/A treatment led to a marked reduction in neutrophils in tumor-bearing mice treated with 0.5 U BoNT/A compared with the untreated tumor group (Figure 4A). Thus, BoNT/A reduced mature neutrophil counts in the peripheral blood of diseased animals while maintaining physiological levels in healthy mice (Figure 4 and Supplementary Figure S2).

As shown in Figure 4B, peripheral blood analysis revealed a decrease in monocyte counts in animals treated with 0.25 U and 0.5 U BoNT/A, probably suggesting that the toxin may limit monocyte recruitment or differentiation into tumor-associated macrophages.

Interestingly, treatment with 1 U BoNT/A did not significantly affect monocyte numbers, which could indicate a dose-dependent tolerance effect associated with the higher initial concentration of the toxin.

Regarding the complete blood count (CBC) analysis (Supplementary Figure S3A–D), no significant changes were detected in erythrocyte count, hemoglobin concentration, lymphocyte count, or platelet number, indicating that the animals maintained an overall good health status. Animals treated with BoNT/A, both with and without tumor induction, showed no alteration in these hematological parameters (Supplementary Figures S2D–G and S3).

These findings demonstrate that the BoNT/A doses used were well tolerated, effectively reducing mammary tumor growth without causing hematological or systemic adverse effects. This contrasts with conventional chemotherapy, which often induces gastrointestinal and hematological toxicity [35], or with radiotherapy, which severely compromises the patient’s health, decreasing their quality of life. Furthermore, administration of BoNT/A to healthy animals produced no statistically significant differences in any hematological parameter tested (Supplementary Figure S2A–G), further confirming the safety and tolerability of the toxin at the evaluated doses.

### 2.3. Effect of BoNT/A on SV2A Expression on Triple-Negative Breast Cancer Tumors

**Figure 5:**
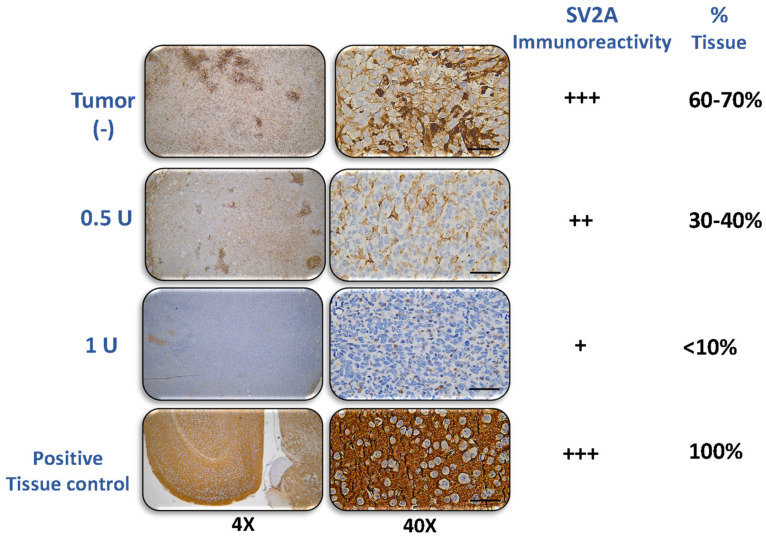
BoNT/A decreased SV2A expression in tumor tissue from the preclinical model of TNBC. No immunoreactivity, + weak, ++ moderate, or +++ intense immunoreactivity, and the percentage of stained cells in the whole tissue area 0–100%. *n* = 3/group; 4× and 40×. Scale bar: 100 μm.

SV2A expression was analyzed in tumor tissues obtained during the progression of the preclinical TNBC model. As the tumor developed, receptor expression at day 24 was predominantly moderate (++), with focal areas of intense (+++) immunoreactivity (Figure 5), covering approximately 60–70% of the total tumor area.

In contrast, tumor tissues from BoNT/A-treated animals showed a marked reduction in SV2A expression. In the group treated with 0.5 U of BoNT/A, SV2A immunoreactivity was moderate (++), observed in about 30–40% of the tumor area. In the 1U treatment group, SV2A expression was weak (+), representing only 10–15% of the total tumor tissue. This decrease suggests a sustained decrease in SV2A levels induced by BoNT/A.

As a positive control, mouse brain tissue derived from healthy experimental mice exhibited intense (+++) immunoreactivity, with 100% of the tissue expressing SV2A (Figure 5). SV2 staining exhibited a neuronal-pattern distribution in the positive control and detectable immunoreactivity in tumor sections, but definitive assignment of SV2A to cancer cells versus neural projections requires higher-resolution and/or co-localization studies.

#### BoNT/A Treatment Did Not Modify the Histological Grade in the 4T1 TNBC Orthotopic Model

According to the Nottingham (Bloom–Richardson) classification, BoNT/A treatment did not modify the histological grade in the preclinical TNBC model. Tumors exhibited poor ductal formation (<10% of total tissue; score 3); marked nuclear pleomorphism with variable size, shape, and dense chromatin (score 3); and 8–14 mitoses per field (score 2). The combined score (8) corresponded to grade III, indicating poorly differentiated tumors. This high-grade morphology persisted in tumors treated with 0.5 U and 1 U of BoNT/A, despite a marked reduction in tumor volume (Supplementary Figure S4).

## 3. Discussion

Triple-negative breast cancer is the most aggressive breast cancer subtype, with a 5-year survival rate of only 10–19% [36,37]. Its care imposes high health costs: annual direct medical per-patient expenses range from USD 20,000 to 300,000 depending on the country and cancer stage, with the highest amounts being for stage IV TNBC [38,39]. Pembrolizumab with chemotherapy and sacituzumab govitecan are the most successful FDA-approved targeted therapies for TNBC, offering significant life and 2.90 quality-adjusted life years (QALY) gains within acceptable costs [40]. Thus, treating PD-L1-positive patients with pembrolizumab plus chemotherapy is cost-effective, offering a 0.70 QALY gain at USD 182,732 compared to chemotherapy alone [41]. Treatment use in patients with metastatic TNBC with olaparib was not considered cost-effective compared with chemotherapy from a Japanese perspective, but it is cost-effective from a US perspective (based on the findings of the OlympiA randomized clinical trial) [42,43]. Talazoparib has not been shown to be a cost-effective option in the treatment of TNBC from a Spanish and German perspective [44,45]. The high costs of current treatments underscore the urgent need for developing new, effective, and affordable therapies for TNBC patients.

Identifying specific biomarkers for targeted cancer therapies remains a major challenge. One promising molecule is SV2A, a protein involved in vesicular release across different cell types; it has emerged as a potential biomarker across various cancer types [46]. This study is the first to report SV2A expression in 4T1 TNBC cells. These findings align with our previous research showing SV2A expression in human breast cancer cell lines MDA-MB-231 and T47D, demonstrating that BoNT/A induced a caspase-3 and -7-dependent apoptotic process in the T47D breast cancer cell line, leading to reduced cell viability [32]. Similar results have been found in prostate human cancer cell lines LnCaP and PC3 [14]. Elevated SV2A levels have been linked to neuroblastoma and brain tumor-related epilepsy [47,48], suggesting its potential use as a biomarker but also as a target in distinct cancer types.

Modulation of SV2A has been shown to be key to neuronal excitability and synaptic transmission; levetiracetam, an antiepileptic drug, binds SV2A to regulate vesicular exocytosis and to suppress abnormal neurotransmitter release [49]. Similarly, brivaracetam, a potent FDA-approved analog of levetiracetam, binds SV2A with much higher affinity, enhancing synaptic vesicle stability and aiding seizure control [50]. BoNT/A, another SV2 binding drug, is a neurotoxin produced by *Clostridium botulinum*, the bacterium responsible for botulism. Its most widespread use is the treatment of cosmetic or medical conditions by causing muscle relaxation or by blocking nerve signals [51,52]. In addition, BoNT/A has also recently been used for the treatment of plaque psoriasis, benign prostatic hyperplasia, and cerebral palsy [53,54,55]. BoNT/A may also inhibit pathways involved in pain and inflammation, which have been shown to be mediated by acetylcholine [33] in tumor-bearing mice. Although BoNT/A might be toxic at high doses, the types used for therapeutic and cosmetic purposes are highly standardized and several-orders-of-magnitude lower than the reported toxic levels. The advantage of using BoNT/A lies in its well-established safety profile, as it has already been approved by the FDA for cosmetic and medical applications. This safety record supports its potential repurposing for the treatment of diseases such as cancer, where the therapeutic benefits may outweigh the potential risks.

Previous studies have shown that BoNT/A, through binding to SV2A, inhibits the proliferation of prostate cancer and breast cancer cell lines [14,32,56]. In this study, we describe the inhibition of cell proliferation in 4T1 cells by BoNT/A, a finding that is consistent with the previously described effects observed in other breast cancer cell lines (MDA-MB-231 and T47D) [31,32]. This effect might be related to BoNT/A’s known mechanism of action, involving the inhibition of vesicular fusion and release through binding to the SV2A receptor, followed by endocytosis and proteolytic cleavage of SNAP-25 (proteins involved in vesicular release) [57]. We also demonstrate that SV2 is expressed in 4T1 cells and tumors, and that BoNT/A dose-dependently inhibits tumor growth in an orthotopic syngeneic mouse model inoculated with 4T1 cells. SV2A overexpression has been previously reported in neuroendocrine tumors [46], but evidence of its expression in diverse cancer types is limited. These results support the possibility of using BoNT/A as a repurposed drug for the treatment of TNBC.

Treatment with BoNT/A significantly reduced SV2A expression in mouse tumors and exerted potent antitumor effects, probably through receptor blockade or internalization. As a proposed mechanistic insight, BoNT/A might be internalized into the cytosol of 4T1 cells, inhibiting vesicular release, and contributing to its therapeutic efficacy. Thus, it will be important to evaluate vesicular release regulation by BoNT/A in this and other cancer models. It is important to note that the proposal to reposition BoNT/A as a potential treatment for TNBC is novel, and no therapeutic control drug was included because this study was exploratory and aimed to evaluate, for the first time, the direct effects of BoNT/A on TNBC cells in a syngeneic model. Since BoNT/A has no established or mechanistically comparable anticancer reference, using a standard drug would not provide a meaningful comparison. However, future studies will aim to compare, side by side, the effects of BoNT/A with known chemotherapeutic drugs.

Besides reducing tumor growth, we observed an effect of BoNT/A on the prevention of splenomegaly. Splenomegaly associated with tumor growth has been previously reported in this preclinical model, and it has been described as a consequence of splenic hyperfunction due to increased granulocyte activity or portal hypertension caused by tumor cell infiltration [58,59]. The observed effect of BoNT/A on tumor size reduction and in the prevention of splenomegaly suggested its involvement in immune function regulation. BoNT/A administration decreased inflammation in mice, decreasing levels of circulating immune cells, including neutrophils and monocytes (Figure 4). Previous studies in the 4T1 TNBC model have demonstrated that 4T1 cells secrete cathepsin C (CTSC), which enzymatically activates neutrophil membrane-bound proteinase 3 (PR3). This activation promotes the processing of interleukin-1β (IL-1β) and triggers NF-κB signaling, upregulating IL-6 and CCL3 expression to enhance neutrophil recruitment. Moreover, the CTSC–PR3–IL-1β axis induces reactive oxygen species (ROS) production and the formation of neutrophil extracellular traps (NETs), which degrade thrombospondin-1 and facilitate metastatic colonization of lung cancer cells [34,60]. A decrease in monocyte number might also be related to decreased monocyte–macrophage recruitment. The SV2 receptor is involved in vesicular release in diverse cell types. The involvement of extracellular vesicles in the recruitment of monocytes to the tumor microenvironment has been previously described in models where they differentiate into macrophages [61]. Additionally, other studies have reported that 4T1 tumor cells promote the polarization to M2 macrophages, which enhance tumor growth, leukocyte infiltration, angiogenesis, and lung metastasis through increased expression of Ki67, HIF-1α, VEGF-A, and CD31 [62].

The long-term effect of BoNT/A could be attributed to its known resistance to proteasomal degradation, as reported by other researchers [57], allowing prolonged activity of the toxin. Importantly, in this study, therapeutic doses of BoNT/A did not compromise animal health, as indicated by stable body weight; normal food intake; and unaltered levels of lymphocytes, erythrocytes, and platelets in peripheral blood. Thus, a safety profile of BoNT/A treatment is warranted to continue its drug-development pathway. This finding is relevant because weight loss has been reported as an indicator of disease progression and recurrence in breast cancer patients [63]. BoNTA is known to be generally well tolerated, and it has been reported that treatment with BoNT/A in 369 patients with spasticity caused adverse effects in only 3% of patients. These effects included general disorders and administration-site conditions (3%), nervous system disorders (1.1%), gastrointestinal disorders (0.3%), infections, and infestations (0.3%) [64]. Also, treatment of refractory chronic migraine with BoNT/A is known to cause lateral eyebrow elevation (19.1%), neck pain (5.3%), and ptosis (4.3%) [65].

**Figure 6:**
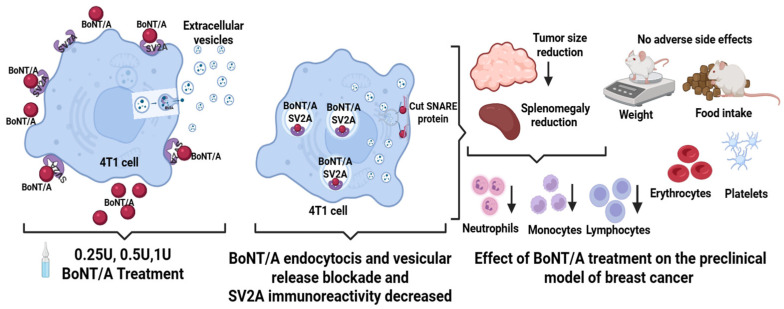
Proposed antitumor effect of BoNT/A through SV2A receptor blockade in a preclinical model of TNBC. After binding SV2, the internalization of BoNT/A into the cytosol of 4T1 cells might lead to the inhibition of vesicular release, reflected by a reduction in tumor growth, and a decrease in inflammation. Treatment with the toxin did not affect health-related parameters, including body weight, food intake, erythrocyte count, or platelet levels in peripheral blood.

Therefore, our results show a potential use of BoNT/A for the treatment of TNBC, a highly aggressive disease whose treatment has been hindered by limited therapeutic options for women with this disease. We have shown that BoNT/A is a safe drug in this model with effective use in cancer positive for SV2, its binding molecule. We propose that the antitumor effect of BONT/A might be mediated through receptor blockade in a preclinical model of TNBC (Figure 6).

One limitation of this study is that the ex vivo weight of tumors and target organs was not recorded. Tumor burden was evaluated longitudinally through vivo measurements, which allowed us to monitor tumor kinetics over time. Obtaining the final tumor and organ weights would have provided additional quantitative information and will further strengthen our future analyses.

BoNT/A demonstrated potential as a therapeutic option for TNBC through its interaction with SV2A and its ability to reduce tumor growth. Nevertheless, further research is essential, as current findings are limited to a murine model and lack safety evaluations, as well as direct comparisons with standard treatments. Considering the high metastatic potential of the 4T1 model, comprehensive evaluation of metastases in distant organs should be incorporated into future investigations. Also, future studies should incorporate survival analyses, safety evaluations, and comprehensive metastatic assessments across multiple tumor models to further support the preclinical development of BoNT/A.

## 4. Conclusions

Our findings reveal SV2A as a novel target which might be overexpressed in TNBC and demonstrate that BoNT/A, very likely through its interaction with SV2A, exerted significant antitumor and immunomodulatory effects without inducing adverse outcomes. BoNT/A repurposing for the treatment of TNBC is particularly attractive given its established clinical use, standardized dosing, and well-documented tolerability, which may facilitate translational development while reducing costs associated with de novo drug discovery. These results support further investigation of BoNT/A as a promising targeted therapeutic strategy for the treatment of TNBC.

## 5. Materials and Methods

### 5.1. Cell Culture

The 4T1 murine triple-negative breast cancer (TNBC) cell line (ATCC^®^ CRL-2539, lot: 700281810, American Type Culture Collection, Manassas, VA, USA) was cultured in RPMI-1640 medium (RPP10-10XLT Caisson, Smithfield, UT, USA) supplemented with 10% Fetal Bovine Serum (FBS, S1560-500, Biowest, MO, USA) and 1% streptomycin/penicillin. The MDA-MB-231 cell line (human TNBC cell line) and U87 cell line (human glioblastoma) were cultured in F12 medium (DFP18-1LT, Caisson, Smithfield, UT, USA) supplemented with 10% FBS and 1% streptomycin/penicillin. EpH4-ev cells (ATCC^®^ CRL-3063, American Type Culture Collection, Manassas, VA, USA, non-tumor mouse mammary cell line) were cultured with DMEM medium (DMP15, Caisson, Smithfield, UT, USA) supplemented with 10% calf serum and 1% streptomycin/penicillin. All cells were incubated at 37 °C and 5% CO_2_.

### 5.2. Western Blot Analysis

4T1 (passage number 15), U87 (ATCC^®^ HTB-14, passage number 45), MDA-MB-231 (ATCC^®^ HTB-26, lot: 70000792, passage number 48), and EpH4-ev cells (passage number 37) with 3 × 10^5^ cells/well were cultured overnight in a six-well plate. The harvested cells were washed with ice-cold phosphate-buffered saline (PBS), and the proteins were extracted as mentioned previously [28]. Primary antibody against SV2A, rabbit monoclonal anti-SV2A antibody (66724, Cell Signalling Technologies, Danvers, MA, USA, recognizing mouse, rat, or human SV2) at a dilution of 1:200 or β-actin/mouse monoclonal anti-ACTIN antibody (A5441, Sigma-Aldrich, Merck Life Science, Estado de Mexico, Mexico) at a dilution 1:5000, was incubated at 4 °C overnight; the corresponding secondary antibodies, Anti-Rabbit IgG-HRP (314661, Thermo Fisher Scientific, Waltham, MA, USA) at a 1:5000 dilution or anti-Mouse IgG-HRP (ab97046, Abcam, Cambridge, UK) at a 1:1000 dilution, were incubated 1 h at room temperature. The blot bands were observed using an ECL Chemiluminescence (Santa Cruz Biotechnology, Dallas, TX, USA), using the LI-COR C-DiGit^TM^ Blot Scanner (LI-COR, Lincoln, NB, USA). Data show the analysis of three independent experiments.

### 5.3. Determination of Cell Viability

The effect of BoNT/A (Dysport) on 4T1 cell viability was determined by 3-(4,5-dimethylthiazol-2-yl)-2,5- diphenyltetrazolium bromide (MTT) assay. Briefly, 3.5 × l0^3^ cells/well were treated with different concentrations of BoNT/A (0–15 U). After a 24 h, 48 h, or 72 h incubation period, the cells were washed twice with PBS, and MTT (5 mg/mL in PBS) was added to each well and incubated at 37 °C for 4 h. Formazan crystals formed and were dissolved by adding dimethyl sulfoxide (DMSO, 100 μL/well), and the absorbance was read at 550 nm, using a microplate reader (Synergy 4, Biotek Model 3550; Richmond, VA, USA). The reduction in cell viability after BoNT/A treatment was expressed in terms of control (non-BoNT/A treated) cells. Percentages of cell survival were calculated as follows: %cell survival = (absorbance of treated cells/absorbance of cells with vehicle) × 100. The half inhibitory concentration (IC_50_) was calculated from the dose–response curve obtained by plotting the percentage of cell survival versus the concentration of BoNT/A. It was analyzed with the Gen 5 software; the mean value was calculated with 3 independent replicates. The unit of BoNT/A from Allergan (Botox^®^, Allergan Aesthetics, AbbVie Inc. North Chicago, IL, USA) does not correspond to a measure of weight but rather to a biological potency unit defined by the manufacturer through a mouse bioassay, in which the amount required to produce a standard effect (LD_50_) is determined. These units are specific to each brand and cannot be interchanged or converted into micrograms, as they depend on biological activity and the reference method used.

### 5.4. Animals and Ethics Statement

All BALB/c mice at 8–9 weeks old with approximately 18–22 g of body weight were obtained from the animal facility at the Centro Médico Nacional Siglo XXI, IMSS. The mice were randomly assigned to control or treatment groups and separately housed in groups of six under specific pathogen-free conditions with a 23 ± 2 °C room temperature, 30–60% humidity, and a 12 h light–dark cycle. The animals had access to regular chow during the entire experiment, Bio-Dieta-Lab (DDL-7100, Abene, Estado de Mexico, Mexico). Every third day, the laboratory animals were weighed using an Ohaus CS5000 compact digital scale, and food consumption was recorded (by weighing food before placing it in the cage and before cleaning). The cleaning of the cages was carried out every third day, along with the replacement of sterile sawdust. The study protocol was approved by two institutions, the first being the Human Ethics Committee 2106 from the Instituto Mexicano del Seguro Social (IMSS), registration number (R-2018-2106-036); and the second being the Animal Ethics Committee of Benemérita Universidad Autónoma de Puebla, protocol number 100251855-UAL-VIEP-22/1 (job number: VIEP/DGI/0707/2022).

### 5.5. Animal Studies

BALB/c mice were anesthetized using a ketamine (100 mg/kg) and xylazine (10 mg/kg) cocktail administered intraperitoneally, followed by inoculation with 4T1 breast cancer 5 × 10^4^ cells (passage number 13–15) with at least 90% viability, resuspended in 50 µL of RPMI medium without FBS, and subcutaneously administrated into the lower left fourth nipple of each experimental individual using insulin syringes (27 G × 13 mm, BD, Columbus, NE, USA). The control group was injected with culture medium free of FBS and without cancer cells. This method has been described by multiple authors, with some variations [62,66,67,68]. A vernier caliper was used daily to record and evaluate the changes in tumor size, together with the palpation method. The following formula was used to define the tumor volumes: T = (L × A^2^)/2 [67]. After 8 days of injection, either 0.25 U, 0.5 U, 1 U BoNT/A, or isotonic saline solution (ISS) was injected in the tumor site. Health monitoring was conducted by measuring body weight and dietary intake every third day. On the 24th day, animals were sacrificed to prevent suffering in accordance with institutional animal care protocols. After euthanasia, primary tumors, tissues, and spleen were excised, cleaned, and photographed on a standardized background, using a 12 MP digital camera at a fixed distance. Uniform LED lighting was used to ensure consistent illumination. Tumors and spleen were measured on a millimeter scale, using a caliper. The morphological changes in breast cancer cells caused by BoNT-A were evaluated via H&E staining. Samples were fixed in formaldehyde for at least 24 h prior to processing after being embedded in paraffin. SV2A expression in tumor was evaluated by immunohistochemistry, as described in Section 5.7. The experimental groups consisted of three intact animals, which we administered the toxin to, and six animals were in the tumor group and the tumor-with-treatment group. After euthanasia, primary tumors were excised, cleaned, and photographed on a standardized background, using a 12 MP digital camera fixed at a constant distance under uniform LED illumination.

### 5.6. Quantification of Peripheral Blood

Blood extraction was performed by cardiac puncture using an insulin syringe (27G × 13 mm), and 500 μL of blood was collected in BD Microtainer tubes with EDTA. The samples were examined in the Medonic M32 (Quinsa, Química Industrial y de la Salud, Mexico City, Mexico) automated hematology equipment. Microscopic analysis of Wright-stained smears was performed from peripheral blood samples from BoNT-A-treated mice. Additionally, the length of the spleens was measured in centimeter using a caliper to determine the degree of splenomegaly.

### 5.7. Histochemistry and Immunohistochemistry

Tissue samples obtained from mice were fixed, paraffin-embedded, and sectioned into 5 μm slices. Histological sections were mounted on slides pretreated with 0.01% (*w*/*v*) Poly-L-lysine (Sigma-Aldrich), then deparaffinized in xylene, and rehydrated through a graded ethanol series. Hematoxylin–eosin staining was performed for general histological evaluation. Tumor tissues were analyzed using a Leica DM 1000 LED optical microscope and Gryphax software V 2.1.0.724 (Jenoptik Optical Systems GmbH, Jena, Germany) to assess tumor aggressiveness. Three parameters were evaluated: presence of ducts, nuclear pleomorphism, and number of mitotic cells per field (40×, 10 fields). Each parameter was scored from 1 to 3, and the total score determined the histological grade: Grade I (3–5 points, well-differentiated), Grade II (6–7 points, poorly differentiated), and Grade III (8–9 points, undifferentiated).

For immunohistochemistry, antigen retrieval was carried out using Novocastra buffer 1× (RE7119, Leica, Wetzlar, Germany) at 100 °C for 1 h. Detection was performed using the Novolink Max Polymer Detection System (Leica RE280-K). Sections were incubated with a primary monoclonal anti-SV2A antibody (mouse, clone E-8; Santa Cruz Biotechnology, sc-376234; is recommended for the detection of SV2A from mouse, rat, and human sources) at a dilution of 1:50. Subsequently, samples were incubated for 2 h at room temperature with a goat anti-mouse IgG secondary antibody conjugated to peroxidase (1:500 dilution in 1% BSA; Ap124P, Chemicon Millipore, Merck Life Science, Mexico City, Mexico). Finally, sections were sealed with mounting medium and visualized under a Leica DM1000 LED optical microscope.

Immunohistochemical (IHC) evaluation was performed by manual counting in 10 randomly selected fields at 40×, calculating the percentage of positive cells. Staining intensity was assessed using a standard semi-quantitative scale (0 = negative, + = weak, ++ = moderate, and +++ = strong). The term “++ with focal areas of +++” indicates predominantly moderate staining with focal regions of high intensity. A positive control was included for reference. Mouse brain tissue was used as a positive control and tissue without primary antibody was used as a negative control [69].

### 5.8. Statistical Analysis

GraphPad Prism 8.0 (GraphPad Software, Inc., La Jolla, CA, USA) was used to perform statistical analysis, in which a one-way or two-way ANOVA followed by Tukey’s post hoc test for multiple comparisons was applied. Shapiro–Wilk test as applied as a normality test. Data are presented as mean ± Standard Error of the Mean (SEM), and * *p* < 0.05 was considered statistically significant.

## Data Availability

The original contributions presented in this study are included in the article/Supplementary Materials. Further inquiries can be directed toward the corresponding author.

## Supplementary Materials

The following supporting information can be downloaded at https://www.mdpi.com/article/10.3390/toxins18050212/s1, Supplementary Figure S1: Effect of BoNT/A on body weight and food intake of control and tumor-bearing mice; Supplementary Figure S2: Effect of BoNT/A on the immune response, no changes were observed in hematological parameters (neutrophils, monocytes, erythrocytes, hemoglobin, platelets and lymphocytes) in non-tumor-bearing mice; Supplementary Figure S3: Analysis of the effect of BoNT/A on the general health status of animals in a preclinical model of triple-negative breast cancer; Supplementary Figure S4: Effect of BoNT/A on histological grade in tumor tissue in a preclinical model of triple negative breast cancer.

## Abbreviations

ANOVA	Analysis of Variance
BALB	Bagg/Albin
BC	Blood Count
BoNT/A	Botulinum Neurotoxin A
CCL3	C-C motif chemokine ligand 3
CD31	Cluster of differentiation 31
CTRL	Control
EC50	Half Maximal Effective Concentration
EV	Extracellular Vesicles
FDA	Food and Drug Administration
HER2	Human Epidermal Growth Factor Receptor 2
HIF-1α	Hypoxia-inducible factor 1 alpha
IL-1b	Interlekin-1b
IL-6	Interlekin-6
LNCaP	Lymph Node Carcinoma of the Prostate
MDA-MB-231	M. D. Anderson-Metastatic Breast-231
MTT	Microculture Tetrazolium, (3-(4,5-dimethylthiazol-2-yl)-2,5-diphenyltetrazolium bromide)
PD-1	Programmed Cell Death Protein 1
PD-L1	Programmed Death-Ligand 1
QALY	Quality-adjusted life years
SEM	Standard Error of the Mean
RPMI	Roswell Park Memorial Institute Medium
SNAP 25	Synaptosomal-Associated Protein 25
SSI	Sterile Saline (Sodium Chloride 0.9%) for Injection
SV2A	Synaptic vesicle glycoprotein 2A
T	Tumor
TMS	Tissue Microscope System
TNBC	Triple-negative breast cancer
TROP-2	Trophoblast Surface Antigen 2
U	Unit
U87	Uppsala 87 Malignant Glioma
VEGF-A	Vascular endothelial growth factor
