# Supplementary material for: Botulinum Neurotoxin-A Inhibits Tumor Growth in a Triple-Negative Breast Cancer Preclinical Model
Source: Toxins (Basel). 2026 Apr 30;18(5):212. doi: 10.3390/toxins18050212 (PMC13211571; doi:10.3390/toxins18050212)
Supplement: Supplementary file 1 [file toxins-18-00212-s001.zip › toxins-4013158-supplementary.pdf]

Supplementary Materials: Botulinum Neurotoxin-A Inhibits Tumor Growth in a Triple-Negative Breast Cancer Preclinical Model

Evoli N. Lopez, Guadalupe Delgado-López, Paola Maycotte, Pablo Hernández-Jáuregui, Irma Herrera-Camacho, Nora Hilda Rosas-Murrieta, Eunice López-Muñoz, Claudia Teresita Gutiérrez-Quiroz, Uriel Ramírez-Carrera, Cindy Bandala, Lourdes Millán-Pérez-Peña and Maricruz Anaya-Ruiz

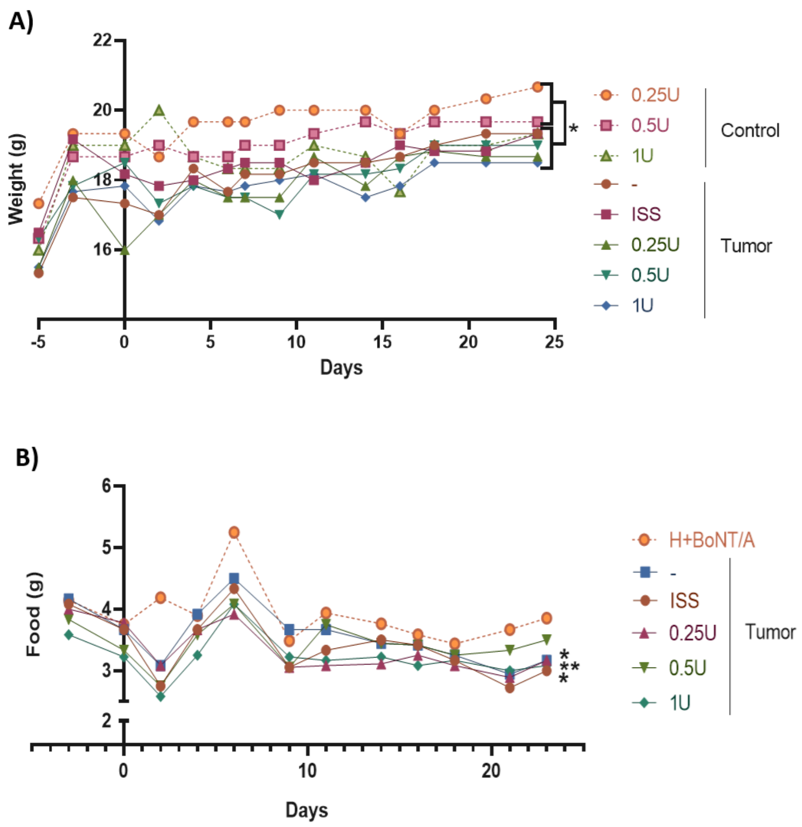

Supplementary Figure S1. Effect of BoNT/A on body weight and food intake of control and tumor-bearing mice. **(A)** Analysis of control and tumor-bearing mice with BoNT/A treatment. The graph show mean +/- SEM, n=6-10, One way ANOVA, Tukey´s post hoc analysis *p< 0.05. **(B)** Food intake of tumor bearing mice. The graph shows statistical differences between tumor-bearing mice (ISS, 0.25U and 1U) with respect to healthy mice without tumor treated with BoNT/A with 0.25U, 0.5U y 1U (H+BoNT/A); (ISS, isotonic saline solution). The graph shows mean +/- SEM n=6-10. One way ANOVA, Tukey´s post hoc analysis * p<0.05; ** p<0.01.

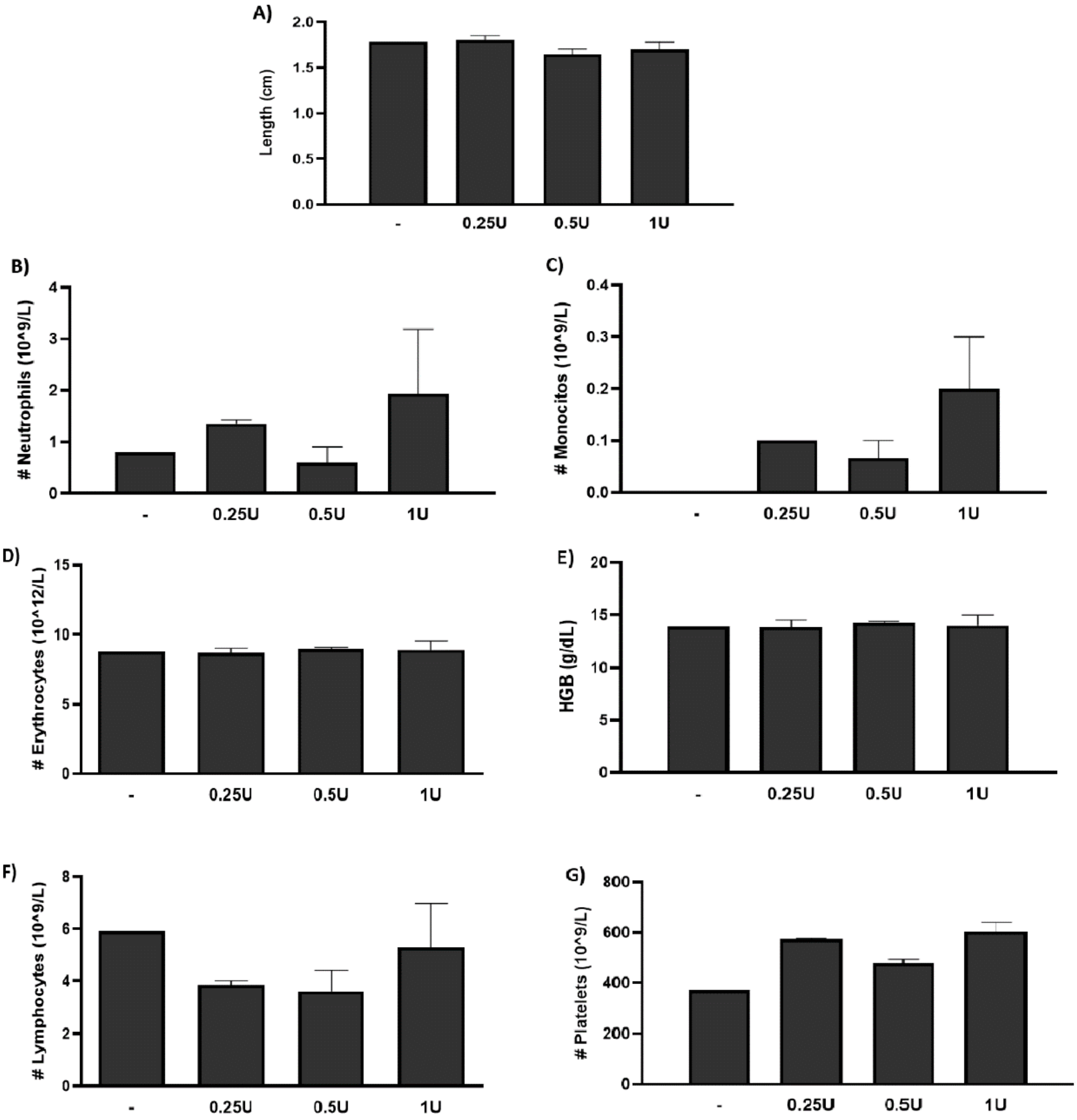

Supplementary Figure S2. Effect of BoNT/A on the immune response, no changes were observed in hematological parameters (neutrophils, monocytes, erythrocytes, hemoglobin, platelets and lymphocytes) in non-tumor-bearing mice. **(A)** No differences in spleen size were observed with treatment; **(B-G)** BoNT/A did not induce statistically significant differences in the immune response and blood parameters evaluated (number of neutrophils, the monocytes, erythrocytes, hemoglobin, lymphocytes and platelets). A-G graphs show mean +/- SEM of 1-3 independent experiments.

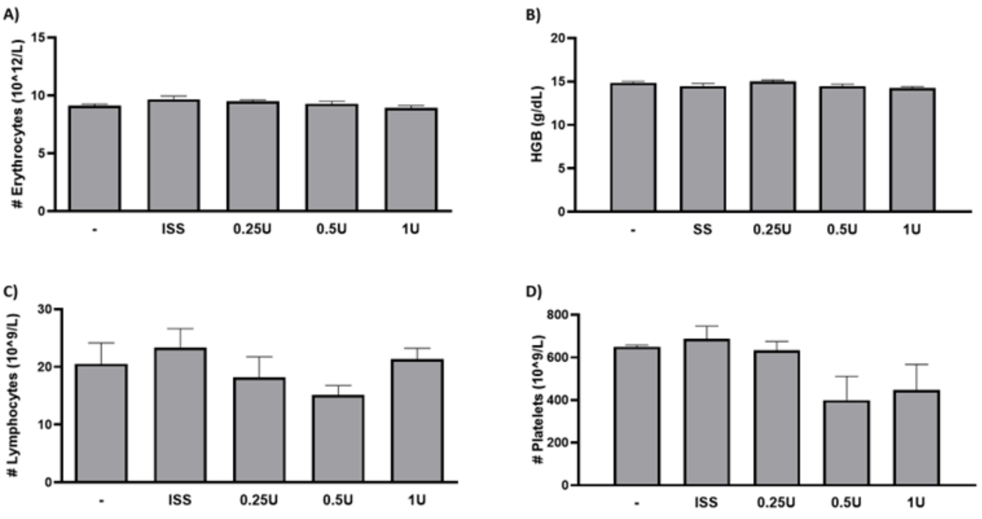

Supplementary Figure S3. Analysis of the effect of BoNT/A on the general health status of animals in a preclinical model of triple-negative breast cancer. **(A-D)** BoNT/A treatment had no effect on erythrocytes, hemoglobin, lymphocytes and platelets of tumor-bearing mice. Graphs show mean +/- SEM of 1-3 independent experiments.

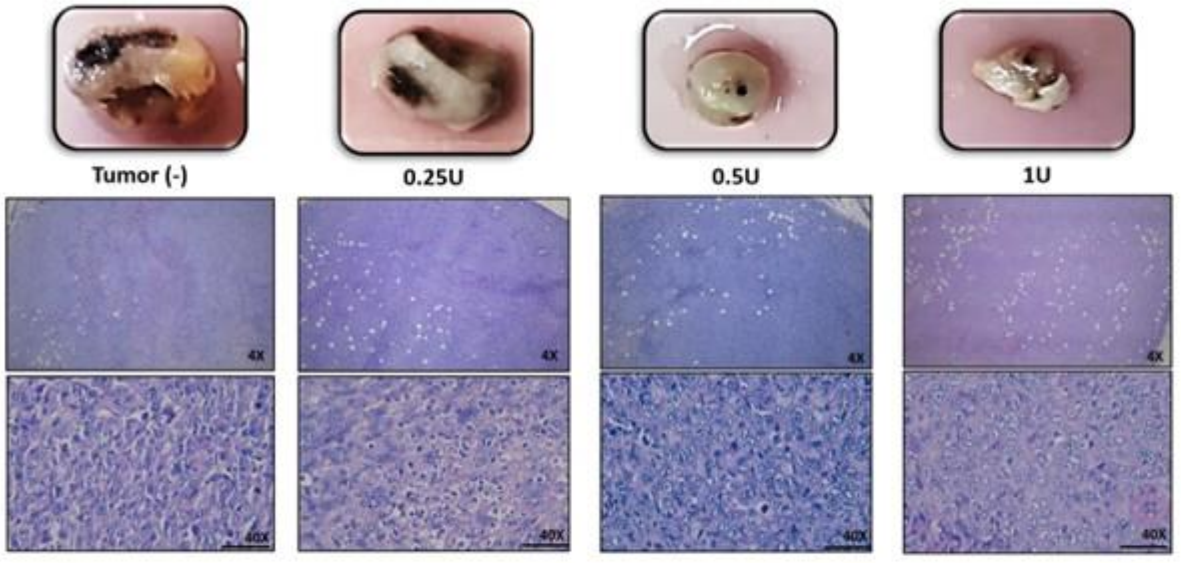

Supplementary Figure S4. Effect of BoNT/A on histological grade in tumor tissue in a preclinical model of triple negative breast cancer. The treatment with 0.5U and 1U of toxin is evidenced, with a poorly differentiated grade III. 4X and 40X amplitude with 3 independent experiments. Scale bar: 100 μm.
